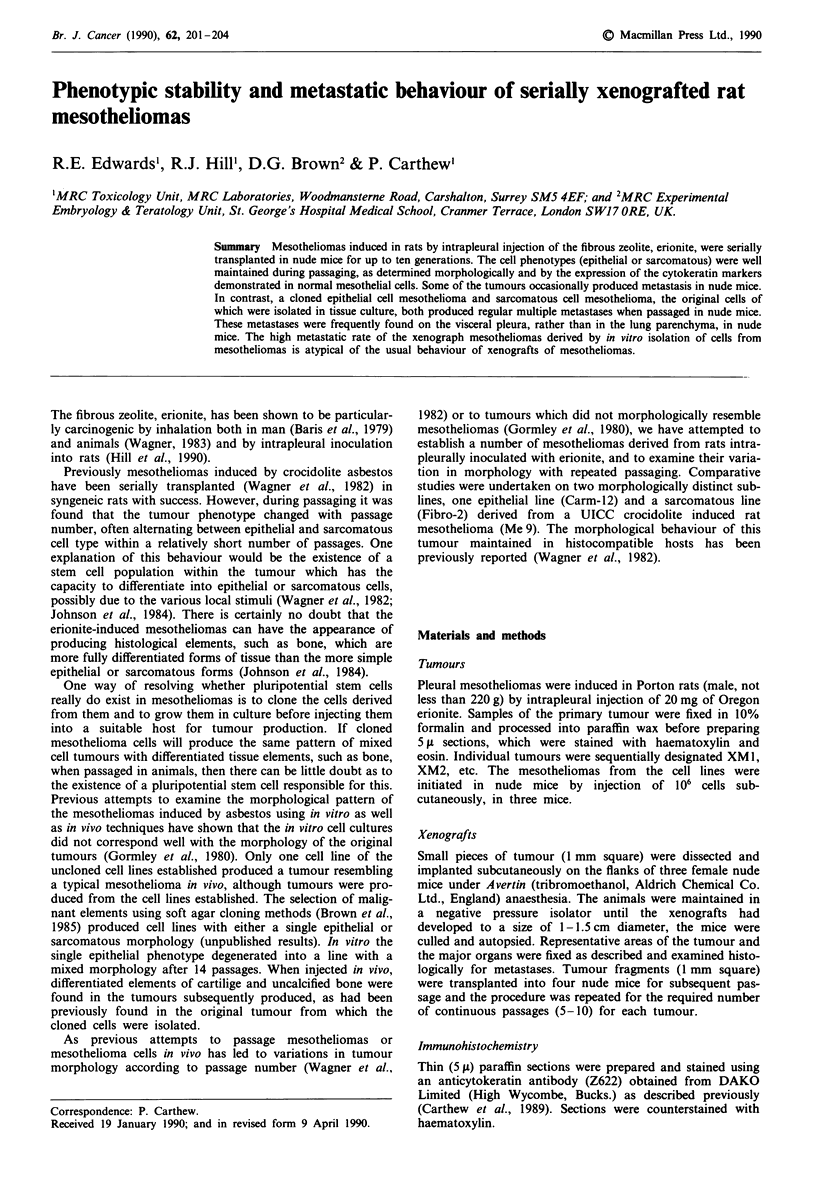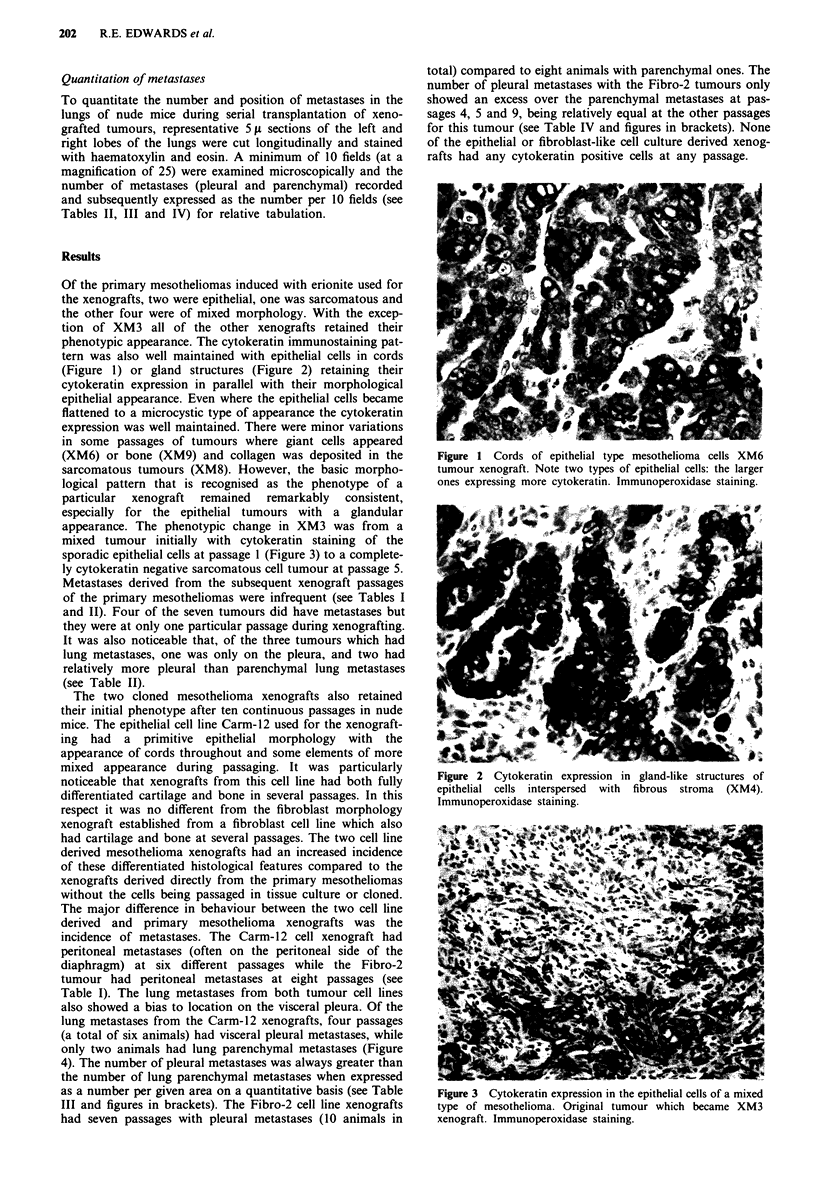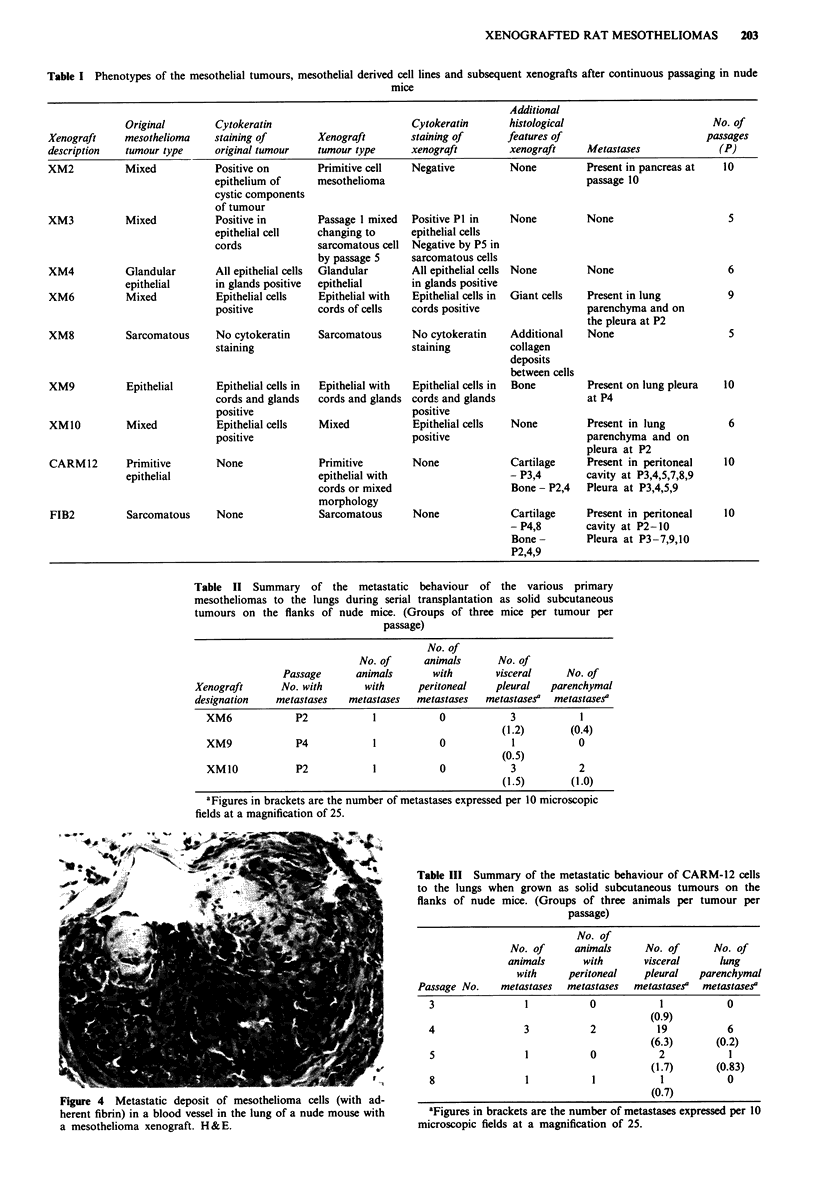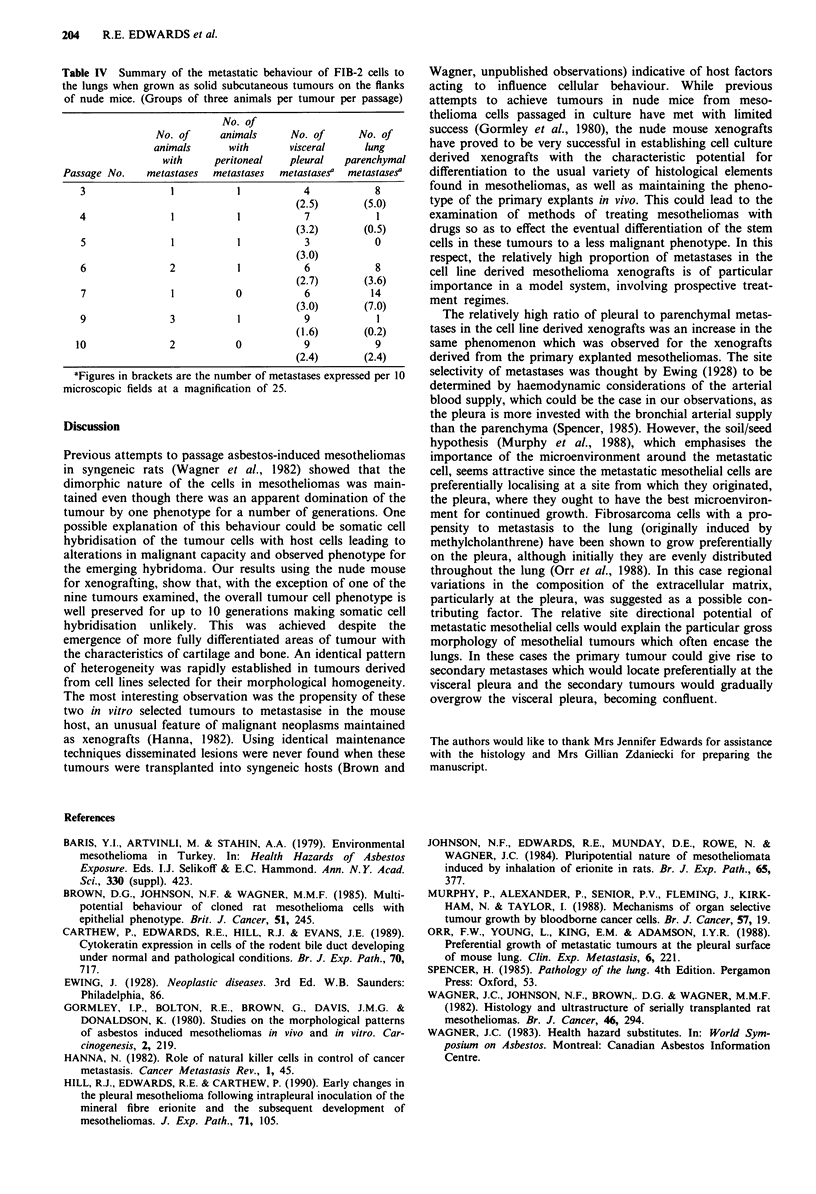# Phenotypic stability and metastatic behaviour of serially xenografted rat mesotheliomas.

**DOI:** 10.1038/bjc.1990.260

**Published:** 1990-08

**Authors:** R. E. Edwards, R. J. Hill, D. G. Brown, P. Carthew

**Affiliations:** MRC Toxicology Unit, MRC Laboratories, Carshalton, Surrey, UK.

## Abstract

**Images:**


Br. J. Cancer (1990), 62, 201-204 © Macmillan Press Ltd., 1990

### Phenotypic stability and metastatic behaviour of serially xenografted rat mesotheliomas

R.E. Edwards[1], R.J. Hill[1], D.G. Brown[2] & P. Carthew[1]

[1]*MRC Toxicology Unit, MRC Laboratories, Woodmansterne Road, Carshalton, Surrey SM5 4EF; and* [2]*MRC Experimental Embryology & Teratology Unit, St. George's Hospital Medical School, Cranmer Terrace, London SW17 0RE, UK.*

**Summary** Mesotheliomas induced in rats by intrapleural injection of the fibrous zeolite, erionite, were serially transplanted in nude mice for up to ten generations. The cell phenotypes (epithelial or sarcomatous) were well maintained during passaging, as determined morphologically and by the expression of the cytokeratin markers demonstrated in normal mesothelial cells. Some of the tumours occasionally produced metastasis in nude mice. In contrast, a cloned epithelial cell mesothelioma and sarcomatous cell mesothelioma, the original cells of which were isolated in tissue culture, both produced regular multiple metastases when passaged in nude mice. These metastases were frequently found on the visceral pleura, rather than in the lung parenchyma, in nude mice. The high metastatic rate of the xenograph mesotheliomas derived by *in vitro* isolation of cells from mesotheliomas is atypical of the usual behaviour of xenografts of mesotheliomas.

The fibrous zeolite, erionite, has been shown to be particularly carcinogenic by inhalation both in man (Baris *et al.*, 1979) and animals (Wagner, 1983) and by intrapleural inoculation into rats (Hill *et al.*, 1990).

Previously mesotheliomas induced by crocidolite asbestos have been serially transplanted (Wagner *et al.*, 1982) in syngeneic rats with success. However, during passaging it was found that the tumour phenotype changed with passage number, often alternating between epithelial and sarcomatous cell type within a relatively short number of passages. One explanation of this behaviour would be the existence of a stem cell population within the tumour which has the capacity to differentiate into epithelial or sarcomatous cells, possibly due to the various local stimuli (Wagner *et al.*, 1982; Johnson *et al.*, 1984). There is certainly no doubt that the erionite-induced mesotheliomas can have the appearance of producing histological elements, such as bone, which are more fully differentiated forms of tissue than the more simple epithelial or sarcomatous forms (Johnson *et al.*, 1984).

One way of resolving whether pluripotential stem cells really do exist in mesotheliomas is to clone the cells derived from them and to grow them in culture before injecting them into a suitable host for tumour production. If cloned mesothelioma cells will produce the same pattern of mixed cell tumours with differentiated tissue elements, such as bone, when passaged in animals, then there can be little doubt as to the existence of a pluripotential stem cell responsible for this. Previous attempts to examine the morphological pattern of the mesotheliomas induced by asbestos using *in vitro* as well as *in vivo* techniques have shown that the *in vitro* cell cultures did not correspond well with the morphology of the original tumours (Gormley *et al.*, 1980). Only one cell line of the uncloned cell lines established produced a tumour resembling a typical mesothelioma *in vivo*, although tumours were produced from the cell lines established. The selection of malignant elements using soft agar cloning methods (Brown *et al.*, 1985) produced cell lines with either a single epithelial or sarcomatous morphology (unpublished results). *In vitro* the single epithelial phenotype degenerated into a line with a mixed morphology after 14 passages. When injected *in vivo*, differentiated elements of cartilige and uncalcified bone were found in the tumours subsequently produced, as had been previously found in the original tumour from which the cloned cells were isolated.

As previous attempts to passage mesotheliomas or mesothelioma cells *in vivo* has led to variations in tumour morphology according to passage number (Wagner *et al.*, 1982) or to tumours which did not morphologically resemble mesotheliomas (Gormley *et al.*, 1980), we have attempted to establish a number of mesotheliomas derived from rats intrapleurally inoculated with erionite, and to examine their variation in morphology with repeated passaging. Comparative studies were undertaken on two morphologically distinct sublines, one epithelial line (Carm-12) and a sarcomatous line (Fibro-2) derived from a UICC crocidolite induced rat mesothelioma (Me 9). The morphological behaviour of this tumour maintained in histocompatible hosts has been previously reported (Wagner *et al.*, 1982).

### Materials and methods

### *Tumours*

Pleural mesotheliomas were induced in Porton rats (male, not less than 220 g) by intrapleural injection of 20 mg of Oregon erionite. Samples of the primary tumour were fixed in 10% formalin and processed into paraffin wax before preparing 5 µ sections, which were stained with haematoxylin and eosin. Individual tumours were sequentially designated XM1, XM2, etc. The mesotheliomas from the cell lines were initiated in nude mice by injection of $10^6$ cells subcutaneously, in three mice.

### *Xenografts*

Small pieces of tumour (1 mm square) were dissected and implanted subcutaneously on the flanks of three female nude mice under *Avertin* (tribromoethanol, Aldrich Chemical Co. Ltd., England) anaesthesia. The animals were maintained in a negative pressure isolator until the xenografts had developed to a size of 1-1.5 cm diameter, the mice were culled and autopsied. Representative areas of the tumour and the major organs were fixed as described and examined histologically for metastases. Tumour fragments (1 mm square) were transplanted into four nude mice for subsequent passage and the procedure was repeated for the required number of continuous passages (5-10) for each tumour.

### *Immunohistochemistry*

Thin (5 µ) paraffin sections were prepared and stained using an anticytokeratin antibody (Z622) obtained from DAKO Limited (High Wycombe, Bucks.) as described previously (Carthew *et al.*, 1989). Sections were counterstained with haematoxylin.

Correspondence: P. Carthew.
Received 19 January 1990; and in revised form 9 April 1990.

*Quantitation of metastases*

To quantitate the number and position of metastases in the lungs of nude mice during serial transplantation of xenografted tumours, representative 5 $\mu$ sections of the left and right lobes of the lungs were cut longitudinally and stained with haematoxylin and eosin. A minimum of 10 fields (at a magnification of 25) were examined microscopically and the number of metastases (pleural and parenchymal) recorded and subsequently expressed as the number per 10 fields (see Tables II, III and IV) for relative tabulation.

### Results

Of the primary mesotheliomas induced with erionite used for the xenografts, two were epithelial, one was sarcomatous and the other four were of mixed morphology. With the exception of XM3 all of the other xenografts retained their phenotypic appearance. The cytokeratin immunostaining pattern was also well maintained with epithelial cells in cords (Figure 1) or gland structures (Figure 2) retaining their cytokeratin expression in parallel with their morphological epithelial appearance. Even where the epithelial cells became flattened to a microcystic type of appearance the cytokeratin expression was well maintained. There were minor variations in some passages of tumours where giant cells appeared (XM6) or bone (XM9) and collagen was deposited in the sarcomatous tumours (XM8). However, the basic morphological pattern that is recognised as the phenotype of a particular xenograft remained remarkably consistent, especially for the epithelial tumours with a glandular appearance. The phenotypic change in XM3 was from a mixed tumour initially with cytokeratin staining of the sporadic epithelial cells at passage 1 (Figure 3) to a completely cytokeratin negative sarcomatous cell tumour at passage 5. Metastases derived from the subsequent xenograft passages of the primary mesotheliomas were infrequent (see Tables I and II). Four of the seven tumours did have metastases but they were at only one particular passage during xenografting. It was also noticeable that, of the three tumours which had lung metastases, one was only on the pleura, and two had relatively more pleural than parenchymal lung metastases (see Table II).

The two cloned mesothelioma xenografts also retained their initial phenotype after ten continuous passages in nude mice. The epithelial cell line Carm-12 used for the xenografting had a primitive epithelial morphology with the appearance of cords throughout and some elements of more mixed appearance during passaging. It was particularly noticeable that xenografts from this cell line had both fully differentiated cartilage and bone in several passages. In this respect it was no different from the fibroblast morphology xenograft established from a fibroblast cell line which also had cartilage and bone at several passages. The two cell line derived mesothelioma xenografts had an increased incidence of these differentiated histological features compared to the xenografts derived directly from the primary mesotheliomas without the cells being passaged in tissue culture or cloned. The major difference in behaviour between the two cell line derived and primary mesothelioma xenografts was the incidence of metastases. The Carm-12 cell xenograft had peritoneal metastases (often on the peritoneal side of the diaphragm) at six different passages while the Fibro-2 tumour had peritoneal metastases at eight passages (see Table I). The lung metastases from both tumour cell lines also showed a bias to location on the visceral pleura. Of the lung metastases from the Carm-12 xenografts, four passages (a total of six animals) had visceral pleural metastases, while only two animals had lung parenchymal metastases (Figure 4). The number of pleural metastases was always greater than the number of lung parenchymal metastases when expressed as a number per given area on a quantitative basis (see Table III and figures in brackets). The Fibro-2 cell line xenografts had seven passages with pleural metastases (10 animals in total) compared to eight animals with parenchymal ones. The number of pleural metastases with the Fibro-2 tumours only showed an excess over the parenchymal metastases at passages 4, 5 and 9, being relatively equal at the other passages for this tumour (see Table IV and figures in brackets). None of the epithelial or fibroblast-like cell culture derived xenografts had any cytokeratin positive cells at any passage.

**Figure 1** Cords of epithelial type mesothelioma cells XM6 tumour xenograft. Note two types of epithelial cells: the larger ones expressing more cytokeratin. Immunoperoxidase staining.

**Figure 2** Cytokeratin expression in gland-like structures of epithelial cells interspersed with fibrous stroma (XM4). Immunoperoxidase staining.

**Figure 3** Cytokeratin expression in the epithelial cells of a mixed type of mesothelioma. Original tumour which became XM3 xenograft. Immunoperoxidase staining.

**Table I** Phenotypes of the mesothelial tumours, mesothelial derived cell lines and subsequent xenografts after continuous passaging in nude mice

| *Xenograft description* | *Original mesothelioma tumour type* | *Cytokeratin staining of original tumour* | *Xenograft tumour type* | *Cytokeratin staining of xenograft* | *Additional histological features of xenograft* | *Metastases* | *No. of passages (P)* |
|---|---|---|---|---|---|---|---|
| XM2 | Mixed | Positive on epithelium of cystic components of tumour | Primitive cell mesothelioma | Negative | None | Present in pancreas at passage 10 | 10 |
| XM3 | Mixed | Positive in epithelial cell cords | Passage 1 mixed changing to sarcomatous cell by passage 5 | Positive P1 in epithelial cells Negative by P5 in sarcomatous cells | None | None | 5 |
| XM4 | Glandular epithelial | All epithelial cells in glands positive | Glandular epithelial | All epithelial cells in glands positive | None | None | 6 |
| XM6 | Mixed | Epithelial cells positive | Epithelial with cords of cells | Epithelial cells in cords positive | Giant cells | Present in lung parenchyma and on the pleura at P2 | 9 |
| XM8 | Sarcomatous | No cytokeratin staining | Sarcomatous | No cytokeratin staining | Additional collagen deposits between cells | None | 5 |
| XM9 | Epithelial | Epithelial cells in cords and glands positive | Epithelial with cords and glands | Epithelial cells in cords and glands positive | Bone | Present on lung pleura at P4 | 10 |
| XM10 | Mixed | Epithelial cells positive | Mixed | Epithelial cells positive | None | Present in lung parenchyma and on pleura at P2 | 6 |
| CARM12 | Primitive epithelial | None | Primitive epithelial with cords or mixed morphology | None | Cartilage – P3,4 Bone – P2,4 | Present in peritoneal cavity at P3,4,5,7,8,9 Pleura at P3,4,5,9 | 10 |
| FIB2 | Sarcomatous | None | Sarcomatous | None | Cartilage – P4,8 Bone – P2,4,9 | Present in peritoneal cavity at P2–10 Pleura at P3–7,9,10 | 10 |

**Table II** Summary of the metastatic behaviour of the various primary mesotheliomas to the lungs during serial transplantation as solid subcutaneous tumours on the flanks of nude mice. (Groups of three mice per tumour per passage)

| *Xenograft designation* | *Passage No. with metastases* | *No. of animals with metastases* | *No. of animals with peritoneal metastases* | *No. of visceral pleural metastases[a]* | *No. of parenchymal metastases[a]* |
|---|---|---|---|---|---|
| XM6 | P2 | 1 | 0 | 3 (1.2) | 1 (0.4) |
| XM9 | P4 | 1 | 0 | 1 (0.5) | 0 |
| XM10 | P2 | 1 | 0 | 3 (1.5) | 2 (1.0) |

[a]Figures in brackets are the number of metastases expressed per 10 microscopic fields at a magnification of 25.

**Figure 4** Metastatic deposit of mesothelioma cells (with adherent fibrin) in a blood vessel in the lung of a nude mouse with a mesothelioma xenograft. H&E.

**Table III** Summary of the metastatic behaviour of CARM-12 cells to the lungs when grown as solid subcutaneous tumours on the flanks of nude mice. (Groups of three animals per tumour per passage)

| *Passage No.* | *No. of animals with metastases* | *No. of animals with peritoneal metastases* | *No. of visceral pleural metastases[a]* | *No. of lung parenchymal metastases[a]* |
|---|---|---|---|---|
| 3 | 1 | 0 | 1 (0.9) | 0 |
| 4 | 3 | 2 | 19 (6.3) | 6 (0.2) |
| 5 | 1 | 0 | 2 (1.7) | 1 (0.83) |
| 8 | 1 | 1 | 1 (0.7) | 0 |

[a]Figures in brackets are the number of metastases expressed per 10 microscopic fields at a magnification of 25.

**Table IV** Summary of the metastatic behaviour of FIB-2 cells to the lungs when grown as solid subcutaneous tumours on the flanks of nude mice. (Groups of three animals per tumour per passage)

| *Passage No.* | *No. of animals with metastases* | *No. of animals with peritoneal metastases* | *No. of visceral pleural metastases*[a] | *No. of lung parenchymal metastases*[a] |
|---|---|---|---|---|
| 3 | 1 | 1 | 4 (2.5) | 8 (5.0) |
| 4 | 1 | 1 | 7 (3.2) | 1 (0.5) |
| 5 | 1 | 1 | 3 (3.0) | 0 |
| 6 | 2 | 1 | 6 (2.7) | 8 (3.6) |
| 7 | 1 | 0 | 6 (3.0) | 14 (7.0) |
| 9 | 3 | 1 | 9 (1.6) | 1 (0.2) |
| 10 | 2 | 0 | 9 (2.4) | 9 (2.4) |

[a]Figures in brackets are the number of metastases expressed per 10 microscopic fields at a magnification of 25.

### Discussion

Previous attempts to passage asbestos-induced mesotheliomas in syngeneic rats (Wagner *et al.*, 1982) showed that the dimorphic nature of the cells in mesotheliomas was maintained even though there was an apparent domination of the tumour by one phenotype for a number of generations. One possible explanation of this behaviour could be somatic cell hybridisation of the tumour cells with host cells leading to alterations in malignant capacity and observed phenotype for the emerging hybridoma. Our results using the nude mouse for xenografting, show that, with the exception of one of the nine tumours examined, the overall tumour cell phenotype is well preserved for up to 10 generations making somatic cell hybridisation unlikely. This was achieved despite the emergence of more fully differentiated areas of tumour with the characteristics of cartilage and bone. An identical pattern of heterogeneity was rapidly established in tumours derived from cell lines selected for their morphological homogeneity. The most interesting observation was the propensity of these two *in vitro* selected tumours to metastasise in the mouse host, an unusual feature of malignant neoplasms maintained as xenografts (Hanna, 1982). Using identical maintenance techniques disseminated lesions were never found when these tumours were transplanted into syngeneic hosts (Brown and Wagner, unpublished observations) indicative of host factors acting to influence cellular behaviour. While previous attempts to achieve tumours in nude mice from mesothelioma cells passaged in culture have met with limited success (Gormley *et al.*, 1980), the nude mouse xenografts have proved to be very successful in establishing cell culture derived xenografts with the characteristic potential for differentiation to the usual variety of histological elements found in mesotheliomas, as well as maintaining the phenotype of the primary explants *in vivo*. This could lead to the examination of methods of treating mesotheliomas with drugs so as to effect the eventual differentiation of the stem cells in these tumours to a less malignant phenotype. In this respect, the relatively high proportion of metastases in the cell line derived mesothelioma xenografts is of particular importance in a model system, involving prospective treatment regimes.

The relatively high ratio of pleural to parenchymal metastases in the cell line derived xenografts was an increase in the same phenomenon which was observed for the xenografts derived from the primary explanted mesotheliomas. The site selectivity of metastases was thought by Ewing (1928) to be determined by haemodynamic considerations of the arterial blood supply, which could be the case in our observations, as the pleura is more invested with the bronchial arterial supply than the parenchyma (Spencer, 1985). However, the soil/seed hypothesis (Murphy *et al.*, 1988), which emphasises the importance of the microenvironment around the metastatic cell, seems attractive since the metastatic mesothelial cells are preferentially localising at a site from which they originated, the pleura, where they ought to have the best microenvironment for continued growth. Fibrosarcoma cells with a propensity to metastasis to the lung (originally induced by methylcholanthrene) have been shown to grow preferentially on the pleura, although initially they are evenly distributed throughout the lung (Orr *et al.*, 1988). In this case regional variations in the composition of the extracellular matrix, particularly at the pleura, was suggested as a possible contributing factor. The relative site directional potential of metastatic mesothelial cells would explain the particular gross morphology of mesothelial tumours which often encase the lungs. In these cases the primary tumour could give rise to secondary metastases which would locate preferentially at the visceral pleura and the secondary tumours would gradually overgrow the visceral pleura, becoming confluent.

The authors would like to thank Mrs Jennifer Edwards for assistance with the histology and Mrs Gillian Zdaniecki for preparing the manuscript.

### References

BARIS, Y.I., ARTVINLI, M. & STAHIN, A.A. (1979). Environmental mesothelioma in Turkey. In: *Health Hazards of Asbestos Exposure*. Eds. I.J. Selikoff & E.C. Hammond. *Ann. N.Y. Acad. Sci.*, **330** (suppl). 423.

BROWN, D.G., JOHNSON, N.F. & WAGNER, M.M.F. (1985). Multipotential behaviour of cloned rat mesothelioma cells with epithelial phenotype. *Brit. J. Cancer*, **51**, 245.

CARTHEW, P., EDWARDS, R.E., HILL, R.J. & EVANS, J.E. (1989). Cytokeratin expression in cells of the rodent bile duct developing under normal and pathological conditions. *Br. J. Exp. Path.*, **70**, 717.

EWING, J. (1928). *Neoplastic diseases*. 3rd Ed. W.B. Saunders: Philadelphia, 86.

GORMLEY, I.P., BOLTON, R.E., BROWN, G., DAVIS, J.M.G. & DONALDSON, K. (1980). Studies on the morphological patterns of asbestos induced mesotheliomas *in vivo* and *in vitro*. *Carcinogenesis*, **2**, 219.

HANNA, N. (1982). Role of natural killer cells in control of cancer metastasis. *Cancer Metastasis Rev.*, **1**, 45.

HILL, R.J., EDWARDS, R.E. & CARTHEW, P. (1990). Early changes in the pleural mesothelioma following intrapleural inoculation of the mineral fibre erionite and the subsequent development of mesotheliomas. *J. Exp. Path.*, **71**, 105.

JOHNSON, N.F., EDWARDS, R.E., MUNDAY, D.E., ROWE, N. & WAGNER, J.C. (1984). Pluripotential nature of mesotheliomata induced by inhalation of erionite in rats. *Br. J. Exp. Path.*, **65**, 377.

MURPHY, P., ALEXANDER, P., SENIOR, P.V., FLEMING, J., KIRKHAM, N. & TAYLOR, I. (1988). Mechanisms of organ selective tumour growth by bloodborne cancer cells. *Br. J. Cancer*, **57**, 19.

ORR, F.W., YOUNG, L., KING, E.M. & ADAMSON, I.Y.R. (1988). Preferential growth of metastatic tumours at the pleural surface of mouse lung. *Clin. Exp. Metastasis*, **6**, 221.

SPENCER, H. (1985). *Pathology of the lung*. 4th Edition. Pergamon Press: Oxford, 53.

WAGNER, J.C., JOHNSON, N.F., BROWN,. D.G. & WAGNER, M.M.F. (1982). Histology and ultrastructure of serially transplanted rat mesotheliomas. *Br. J. Cancer*, **46**, 294.

WAGNER, J.C. (1983). Health hazard substitutes. In: *World Symposium on Asbestos*. Montreal: Canadian Asbestos Information Centre.